# Paramedic Endotracheal Intubation Success Rates Before and After an Intensive Airway Management Education Session

**DOI:** 10.7759/cureus.27781

**Published:** 2022-08-08

**Authors:** Alix Carter, Jan L Jensen, Mark Walker, Yves Leroux, Mikiko Terashima, Jennifer McVey

**Affiliations:** 1 Emergency Medicine, Dalhousie University, Halifax, CAN; 2 Nova Scotia, Emergency Health Services, Halifax, CAN; 3 Community Health & Epidemiology, Dalhousie University, Halifax, CAN

**Keywords:** intubation, airway management, emergency medical services, paramedic, allied health personnel

## Abstract

Introduction

Advanced airway management by paramedics is potentially life-saving, but carries a significant risk to patient safety and can be associated with poor clinical outcome if performed incorrectly. Previously, our team had found that an intensive education intervention demonstrated an improvement in paramedic performance on a written exam and increased confidence in airway skills. This study measured intubation success and the number of attempts per patient before and after intensive paramedic airway management education intervention.

Methods

A 10-hour mandatory course was taken by all advanced life support (ALS) paramedics in a provincial system (2009/04-07, n=~395). The course was done during semi-annual continuing education Emergency Health Services (EHS) in-services. These day-long courses were held in person over four months. The electronic charting database was queried for intubation attempts and successful placements 12 months before the training, during the four months of training, and 12 months post-training. The primary outcome is the difference in success rates between the before (pre-intervention) and after (post-intervention) periods. The secondary outcome is the number of attempts per patient. Stationarity of success in pre- and post-periods was tested. The model was fit tested using Maximum Likelihood regression, and variables were tested using the Wald test.

Results

A sample size of 476 intubation attempts in each of the pre- and post-periods was required to detect a 10% improvement with the pre-intervention success of 60%. A total of 1421 intubation attempts occurred; 674 pre-intervention, 604 post-intervention, and 143 during teaching. Seven attempts were excluded (success unknown). Intubation success rates improved, from 0.68 (95% CI 0.64-0.71) to 0.75 (95% CI 0.72-0.78); a difference of 0.076 (95% CI 0.03-0.12) (p = 0.001). Intubation success rates in the pre-intervention and post-intervention periods were found to be static. A significant decrease was found in the number of attempts per patient in the post-period (p = 0.005).

Conclusion

Intubation success increased from 68% to 75% and was maintained over the 12-month post-period. There is a potential that judgment may also have improved, based on the decreased number of attempts per patient. Limitations include missing values, paramedics’ self-reported number of attempts, and the definition of what is considered to be an attempt. In addition to previously demonstrated improvements in paramedic exam and scenario performance, this airway education intervention appears to have made a significant improvement to patient outcomes. These findings support the value of such education interventions to improve performance.

## Introduction

Endotracheal intubation (ETI) has long been considered the gold standard in airway management in acutely ill or injured patients and is one of the hallmark skills of the advanced care paramedic (ACP) [1,2]. It is important to note that recent studies looked into utilizing alternative airway devices to ETI, are resulting in a shift of this gold standard view of ETI [3,4]. The use of advanced airway management has the potential to save lives but can result in poor clinical outcomes. The role of prehospital ETI has been called into question by research, citing poor patient management, unacceptably low success rates, and negative outcomes [1, 5-14]. The lack of techniques to manage the difficult airway, limited training, and continuing education have been identified as areas of concern. In response to this, some have cautioned against blanket decisions to remove airway interventions from the paramedic scope [15]. Rather, they urged an increased emphasis on training, paramedic decision-making, and patient selection as potentially effective strategies for improving the outcomes of patients requiring prehospital airway management [16-18]. There has been an increasing focus on the role of cognition and decision-making in paramedic-delivered care, particularly for high-risk, complex interventions such as advanced airway management [19,20].

In response to this, and relatively poor intubation success rates in our system at an overall success rate of 71% over the year previous to delivery of the course (April 2008-April 2009), all advanced life support (ALS) paramedics in the province of Nova Scotia completed an intensive airway management training course, the *Airway Interventions and Management in Emergencies (AIME)* course, with the goal of improving paramedic knowledge, skills, and decision-making [6,21].

The objective of this study is to determine differences in intubation success rates before (pre-intervention) and after (post-intervention) the *AIME* course. The hypothesis is that intubation success will improve after completion of the mandatory training. This improvement will be different from any pre-existing trend of improving success and will remain above the level of success measured in the “before”* *period, despite any potential for skill degradation over the 12 months following completion of the training.

The abstract of this study was previously presented at the National Association of Emergency Medical Services (EMS) Physicians annual conference held in Tucson, Arizona in January 2012.

## Materials and methods

Training intervention

The *AIME *course was developed by physicians with emergency medicine and anesthesia backgrounds and provides “case-based, hands-on” airway management education for healthcare providers with acute care responsibilities [8]. This course is a 10-hour session that includes didactic and simulation models. Training emphasizes the clinical decision-making points involved in airway management and the practice of specific skills. Using real-life scenarios, the course reinforces the knowledge and psychomotor skills required in airway emergencies. High fidelity manikins were utilized with difficult airway scenarios, meaning multiple challenges, in an attempt to account for real-world prehospital scenarios. The course was executed with the expertise and guidance of the physician authors of AIME, and field training paramedics and YL tailored the course to our system. The *AIME* program was taken by all ALS paramedics in our provincial emergency medical services (EMS) system in mandatory continuing education sessions, delivered from April to July 2009. This study was approved by the Nova Scotia Health research ethics board (REB file #: CDHA-RS/2013-145).

Study setting and population

In Nova Scotia, the scope of ALS paramedics includes the following advanced airway management interventions: extraglottic devices, ETI, and cricothyroidotomy [22]. ALS paramedics in the province also have access to continuous positive airway pressure (CPAP), while air critical care paramedics (CCPs) have rapid sequence intubation (RSI) in their scope. The term “ALS” includes intermediate care paramedics (ICP), ACPs, and CCPs [23].

Data collection

Airway management information is documented by paramedics in the electronic patient care record (ePCR) and is routinely collected by the quality assurance program in their airway registry embedded in the ePCR system. The airway registry was queried for the number of patients in which intubation was attempted and the number of patients in which it was successful, for 12 months prior to the start of the course (the “before” period), during the four months it took to teach the course (the “during” period), and 12 months following the end of the course (the “after” period). The airway registry was also queried for patient descriptors, including age, gender, indication for airway management, and patient presentation.

Outcomes

The primary outcome measure is the difference between the intubation success in the before and after periods, i.e., whether the slope of the line during the training is different from 0. Secondary outcome measures include whether the intubation success changed within the before period, during the after period and after it stabilizes post-intervention. Intubation attempts per patient in each period were also examined. A sub-analysis was conducted based on the categorization of whether the patient was in cardiac arrest (no pulse) and whether they were of a medical or trauma cause.

Analysis

The data were analyzed as monthly mean success in each period: before (12 months), during (four months), and after (12 months) training. The stationarity of each period was determined, where t0 represents the point at which the training began and t1 is the point at which the training ends and the success rate stabilizes. This was modeled using a traditional likelihood method. Three variables are of interest in the model: the rate of change in the before period, the rate of change during the training, and the rate of change in the after period. The modelling equation was: P(S) = {B1t t < t0, B2t t0<=t < t1, B3t t>=t1}. The model was fit using Maximum Likelihood methods, and the variables were tested using the Wald test. B1 and B3 were tested for significance, i.e., if the success rate was stable before and after training. The subsequent step was to test if the training was effective, which was dependent on the results of the first two tests. The difference in intubation success and number of attempts between the before and after periods was tested with an independent samples t-test for data found to be normally distributed and a Mann-Whitney U test for skewed data.

Sample size

The before period success rate was known to be 60%. To detect a 10% improvement with alpha = 0.05 and beta = 0.9, a sample of 476 discrete episodes was required in the before and after periods.

## Results

A total of 1421 discrete intubation episodes occurred during the study period: 674 in the before period, 604 in the after period, and 143 during training. Due to missing data from the success field, seven (three before, two during, and two after) episodes were excluded. The two episodes were excluded from the after period due to the inability to determine whether an intubation was medical or trauma, and whether those were with a pulse or a cardiac arrest. The included patients in each time period were similar, see Table 1.

**Table 1:** Patient demographics and first pass success.

	Before (n = 674)	During (n = 143)	After (n = 604)	Missing data (n = 7)
Age years (SD)	64.10 (18.34)	60.06 (19.45)	62.66 (18.07)	59.80 (15.06)
Female No. (%)	227 (33.68%)	44 (30.56%)	197 (32.72%)	<5
First pass intubation success	0.68 (95% CI 0.64-0.71)	n/a	0.75 (95% CI 0.72-0.78)	n/a

**Figure 1:**
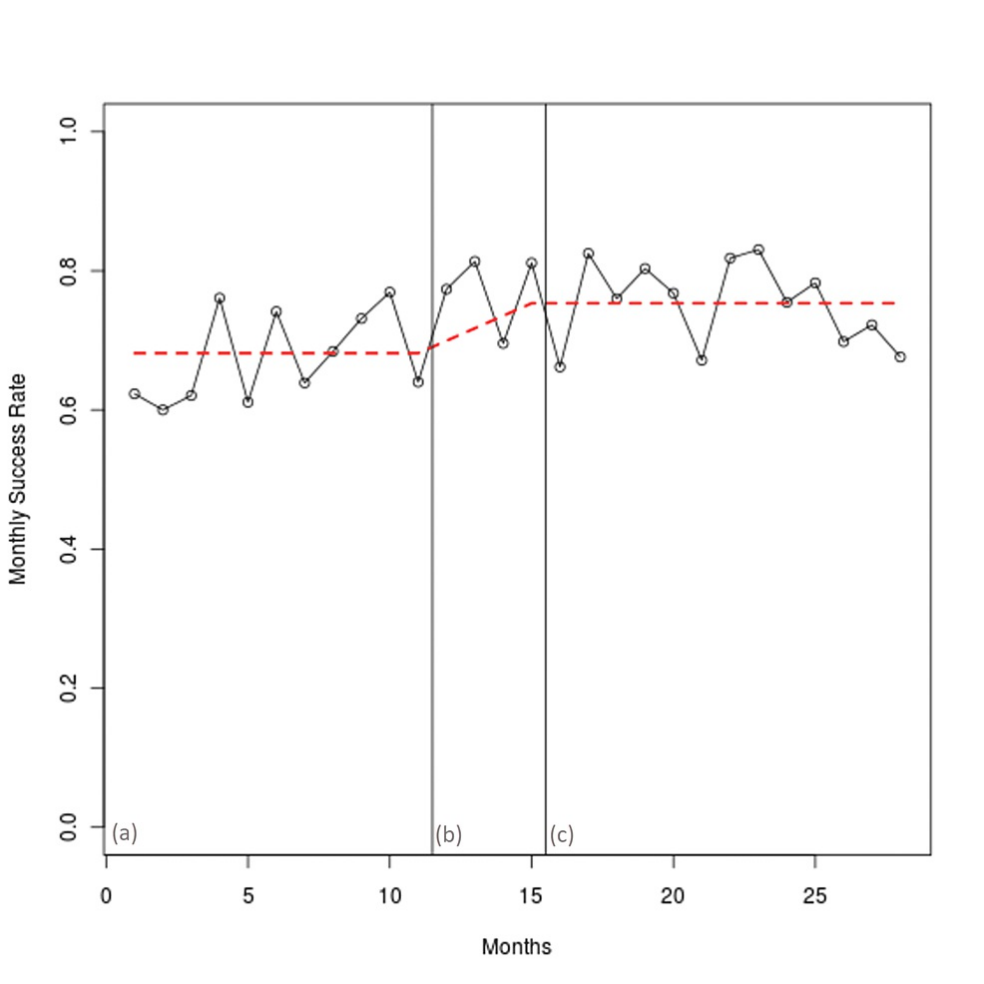
Intubation success rates per study period. Periods are denoted as follows: (a) 12 months pre-intervention, (b) 4 months training, and (c) 12 months post-intervention.

Three regression models were tested, with model two being the best fit, in which the slope or coefficient of the success in the before period and in the after period was not different from zero, or the success was stationary. The coefficient training period is significant and represents an improvement in intubation success during the intervention. Figure 1 demonstrates stable intubation success in the before period, an improvement during the training period, and maintenance of the new stable success for 12 months in the after period.

Success was found to be a normally distributed variable. The average success before the intervention was 0.674 (95% CI 0.64-0.71) and after the intervention, it was 0.75 (95% CI 0.72-0.78). The difference between the two is therefore 0.076 (95% CI 0.03-0.12); a significant improvement (p = 0.001).

**Figure 2:**
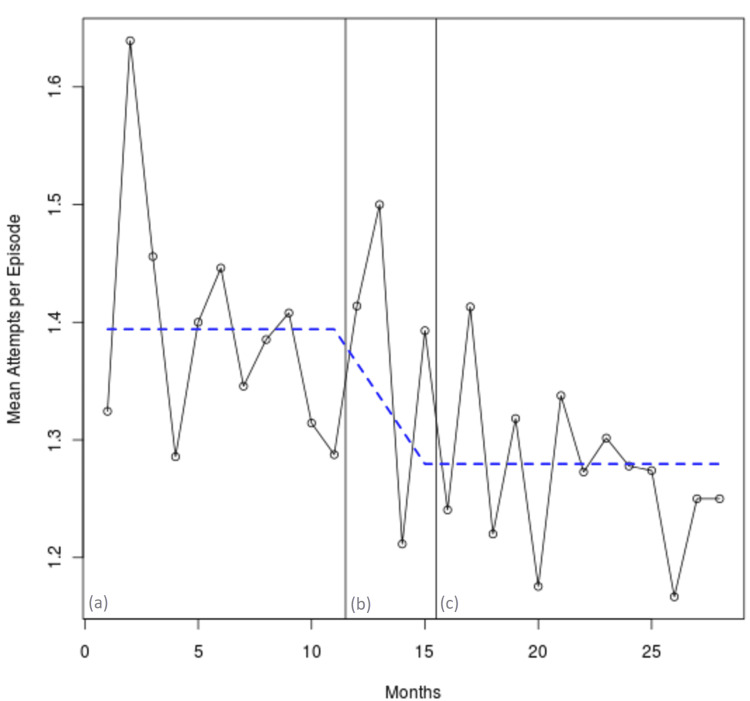
Intubation attempts per patient in each study period. Periods are denoted as follows: (a) 12 months pre-intervention, (b) 4 months training, and (c) 12 months post-intervention.

The number of intubation attempts per patient was found to be left-skewed. The rates were found to be stable in each of the before and after periods. A significant decrease in attempts per patient is noted, from 1.40 to 1.27 (p = 0.005), as shown in Figure 2.

The regression model was repeated for both medical cardiac arrest and medical with a pulse. The numbers in both trauma subgroups are too small for analysis, as can be seen in Table 2. For the medical cardiac arrest subgroup, the success rate before (0.789; 95% CI 0.738-0.840) was not significantly different from the success after (0.843; 95% CI 0.794-0.891) with the difference (0.054; 95% CI -0.010-0.120; p = 0.110). However, the number of attempts per patient decreased post-intervention (p = 0.003).

**Table 2:** A sub-analysis of patients by the presence/absence of a pulse, and categorization into medical or trauma complaints.

Final categorization	Before	During	After
Medical cardiac arrest	497	104	440
Medical with pulse	146	28	119
Trauma cardiac arrest	10	5	15
Trauma with pulse	21	6	30

In the medical with pulse category, the success rates were significantly different between before and after intervention: Before: 0.609 (0.505, 0.713); After: 0.766 (0.676, 0.855); Difference: 0.155 (0.027, 0.386); p = 0.020. However, attempt rates did not see significant improvement. It is the opposite of the medical cardiac arrest category, where success rates did not significantly differ and attempt rates significantly decreased in the group.

## Discussion

This study demonstrates that teaching a hands-on, critical-thinking airway course to paramedics leads to a significant improvement in intubation success. Moreover, the methods of this study tested the stationarity of monthly intubation success over a 12-month time period before and after the intervention, as opposed to simply calculating an average for the entire pre- and post-periods. The strength of this study allows confidence that the intervention was in fact the explanation for the improvement, as the rate was demonstrated to be stationary in the 12 months prior, and importantly, that this improvement has been maintained over the 12 months following the training. This is important to note in light of several prior studies that have shown that psychomotor skills and knowledge used infrequently tends to decay over time [19,24]. Perhaps more importantly, this is important in terms of demonstrating the value of the time, effort, and dollars expended to deliver such training to a large number of paramedics. Similarly, De Lorenzo and Abbot found continuing education in military emergency medical technicians (EMTs) improved skill performance after training and also resulted in gradual improvements over time [16].

The improvement in success rate is driven by the subgroup of medical with a pulse chief complaints, while the medical cardiac arrest group saw an improvement in attempts per patient although no change in overall success. The success in the medical cardiac arrest group was, however, 0.789 before the intervention compared to the success in the whole group at 0.674. Arguably an improvement, chiefly in the subgroup with a pulse (i.e., not cardiac arrest), is a particularly desirable finding in that these would represent the more difficult intubations in the field, especially in the absence of neuromuscular blocking agents. The success in this group was quite low before the intervention, at 0.609.

Also of interest is the finding of fewer intubation attempts per patient in the after period. The *AIME* course is about more than just the psychomotor act of passing an endotracheal tube; it focused on a judgment about determining a difficult airway, when not to attempt intubation, and how to make the first attempt the best. It also encourages clinicians to consider early airway optimization techniques such as re-positioning, and/or using devices such as a bougie and extraglottic device. The finding of fewer intubation attempts per patient in the after period is driven largely by the decrease in attempts in the medical no pulse category. It is possible that another factor here is the emphasis on good chest compressions, and the early consideration of an extraglottic device may be even further enhanced by this additional factor.

Limitations

This study is limited by the self-reported nature of intubation attempts and successes. It is possible that the paramedic will under-report the number of attempts. Also, as a definition of what constitutes an “attempt” was not included within the paramedics’ clinical protocols, it is possible that not all paramedics have the same definition of an “attempt,” which may introduce some uncertainty in the data around the number of attempts. Intubation success became a mandatory field in the charting system part-way through the after period. Therefore, data were incomplete due to this missing field prior to that change. We did not collect data regarding endotracheal tube placement confirmation due to not having access to the hospital records at the time, and it is possible that a small number of these “successes” were not correctly placed in the trachea initially, or subsequently became displaced. We also do not know the impact on neurologically intact survival due to the inability to access hospital records for this data. Finally, in the subgroup analysis, the number of intubations of trauma patients is too small to comment with certainty on the validity of these findings in this population.

## Conclusions

Although ETI has been a key skill of ACPs, previous research has questioned its widespread use and patient benefit. Nova Scotia ALS paramedics were required to participate in the *AIME* course with the goal of improving the first pass success rate of paramedics in the province. Delivery of this intensive airway education program, focusing not only on the technical skill but also on clinical judgment and management of the difficult airway, is shown in this study to improve intubation success in the field. These results were particularly demonstrated in the subgroup of patients with a chief complaint of a medical nature, who were not in cardiac arrest. Educational intervention is key in the positive process measures observed in this study.

## References

[1] Bochicchio GV, Scalea TM (2003). Is field intubation useful?. Curr Opin Crit Care.

[2] Burton JH (2006). Out-of-hospital endotracheal intubation: half empty or half full?. Ann Emerg Med.

[3] Carlson JN, Wang HE (2020). Optimal airway management in cardiac arrest. Crit Care Clin.

[4] Schalk R, Meininger D, Ruesseler M (2011). Emergency airway management in trauma patients using laryngeal tube suction. Prehosp Emerg Care.

[5] Cudnik MT, Newgard CD, Wang H, Bangs C, Herrington R 4th (2008). Distance impacts mortality in trauma patients with an intubation attempt. Prehosp Emerg Care.

[6] Wang HE, Lave JR, Sirio CA, Yealy DM (2006). Paramedic intubation errors: isolated events or symptoms of larger problems?. Health Aff.

[7] Gausche M, Lewis RJ, Stratton SJ (2000). Effect of out-of-hospital pediatric endotracheal intubation on survival and neurological outcome: a controlled clinical trial. JAMA.

[8] Kovacs G, Law A (2022). AIME. Airway Interventions & Management in Emergencies. https://aimeairway.ca/.

[9] Davis DP, Douglas DJ, Koenig W, Carrison D, Buono C, Dunford JV (2007). Hyperventilation following aero-medical rapid sequence intubation may be a deliberate response to hypoxemia. Resuscitation.

[10] Davis DP, Fisher R, Buono C (2006). Predictors of intubation success and therapeutic value of paramedic airway management in a large, urban EMS system. Prehosp Emerg Care.

[11] Wang HE, Yealy DM (2006). How many attempts to accomplish out-of-hospital endotracheal intubation?. Acad Emerg Med.

[12] Murray JA, Demetriades D, Berne TV (2000). Prehospital intubation in patients with severe head injury. J Trauma.

[13] Benger JR, Kirby K, Black S (2018). Effect of a strategy of a supraglottic airway device vs tracheal intubation during out-of-hospital cardiac arrest on functional outcome: the AIRWAYS-2 randomized clinical trial. JAMA.

[14] Hasegawa K, Hiraide A, Chang Y, Brown DF (2013). Association of prehospital advanced airway management with neurologic outcome and survival in patients with out-of-hospital cardiac arrest. JAMA.

[15] Davis DP (2008). Should invasive airway management be done in the field?. CMAJ.

[16] De Lorenzo RA, Abbott CA (2007). Effect of a focused and directed continuing education program on prehospital skill maintenance in key resuscitation areas. J Emerg Med.

[17] Kovacs G, Bullock G, Ackroyd-Stolarz S, Cain E, Petrie D (2000). A randomized controlled trial on the effect of educational interventions in promoting airway management skill maintenance. Ann Emerg Med.

[18] Davis DP, Buono C, Ford J, Paulson L, Koenig W, Carrison D (2007). The effectiveness of a novel, algorithm-based difficult airway curriculum for air medical crews using human patient simulators. Prehosp Emerg Care.

[19] Wang HE, Katz S (2007). Cognitive control and prehospital endotracheal intubation. Prehosp Emerg Care.

[20] Mausz J, Donovan S, McConnell M, Lapalme C, Webb A, Feres E, Tavares W (2017). Reformulations of practice: beyond experience in paramedic airway management. CJEM.

[21] Walker M, Jensen JL, Leroux Y, McVey J, Carter AE (2013). The impact of intense airway management training on paramedic knowledge and confidence measured before, immediately after and at 6 and 12 months after training. Emerg Med J.

[22] (2022). EHS 6200.05 Adult Airway Management. https://novascotia.ca/dhw/ehs/documents/CPG/EHS6200.05%20Adult%20Airway%20Management.pdf.

[23] (2022). National Occupational Competency Profile. http://www.paramedic.ca/site/nocp.

[24] Su E, Schmidt TA, Mann NC, Zechnich AD (2000). A randomized controlled trial to assess decay in acquired knowledge among paramedics completing a pediatric resuscitation course. Acad Emerg Med.

